# RV adaptation to increased afterload in congenital heart disease and pulmonary hypertension

**DOI:** 10.1371/journal.pone.0205196

**Published:** 2018-10-24

**Authors:** Mieke M. P. Driessen, Tim Leiner, Gertjan Tj Sieswerda, Arie P. J. van Dijk, Marco C. Post, Mark K. Friedberg, Luc Mertens, Pieter A. Doevendans, Repke J. Snijder, Erik H. Hulzebos, Folkert J. Meijboom

**Affiliations:** 1 Department of Cardiology, University Medical Centre Utrecht, Utrecht, the Netherlands; 2 ΙCΙN-Netherlands Heart Institute, Utrecht, the Netherlands; 3 Department of Radiology, University Medical Centre Utrecht, Utrecht, the Netherlands; 4 Department of Cardiology, Radboud University Medical Centre Nijmegen, Nijmegen, the Netherlands; 5 Department of Cardiology, Antonius Hospital, Nieuwegein, the Netherlands; 6 Department of Paediatric Cardiology, Labatt Family Heart Center, Toronto, Canada; 7 Department of Pulmonology, Antonius Hospital, Nieuwegein, the Netherlands; 8 Department of Paediatric Physical Therapy and Exercise Physiology, Wilhelmina Children's Hospital, Utrecht, the Netherlands; Cincinnati Children's Hospital Medical Center, UNITED STATES

## Abstract

**Background:**

The various conditions causing a chronic increase of RV pressure greatly differ in the occurrence of RV failure, and in clinical outcome. To get a better understanding of the differences in outcome, RV remodeling, longitudinal function, and transverse function are compared between patients with pulmonary stenosis (PS), those with a systemic RV and those with pulmonary hypertension (PH).

**Materials and methods:**

This cross-sectional study prospectively enrolled subjects for cardiac magnetic resonance imaging (CMR), functional echocardiography and cardiopulmonary exercise testing. The study included: controls (n = 37), patients with PS (n = 15), systemic RV (n = 19) and PH (n = 20). Statistical analysis was performed using Analysis of Variance (ANOVA) with posthoc Bonferroni.

**Results:**

PS patients had smaller RV volumes with higher RV ejection fraction (61.1±9.6%; p<0.05) compared to controls (53.8±4.8%). PH and systemic RV patients exhibited dilated RVs with lower RV ejection fraction (36.9±9.6% and 46.3±10.1%; p<0.01 versus controls). PH patients had lower RV stroke volume (p = 0.02), RV ejection fractions (p<0.01) and VO_2_ peak/kg% (p<0.001) compared to systemic RV patients. Mean apical transverse RV free wall motion was lower and RV free wall shortening (p<0.001) was prolonged in PH patients–resulting in post-systolic shortening and intra-ventricular dyssynchrony. Apical transverse shortening and global longitudinal RV deformation showed the best correlation to RV ejection fraction (respectively r = 0.853, p<0.001 and r = 0.812, p<0.001).

**Conclusions:**

RV remodeling and function differed depending on the etiology of RV pressure overload. In contrast to the RV of patients with PS or a systemic RV, in whom sufficient stroke volumes are maintained, the RV of patients with PH seems unable to compensate for its increase in afterload completely. Key mediators of RV dysfunction observed in PH patients, were: prolonged RV free wall shortening, resulting in post-systolic shortening and intra-ventricular dyssynchrony, and decreased transverse function.

## Introduction

The normal right ventricle (RV) works at low pressures and is believed to tolerate an increase in afterload poorly. This is undoubtedly true for a *sudden* rise in afterload, as present in massive pulmonary embolism, which leads to an immediate decrease in stroke volume and cardiac output. [1] In the case of chronic elevations in RV pressure, this cause-and-effect relationship is less straightforward. Comparing pulmonary hypertension (PH), acquired later in life, to several congenital heart defects (CHD), present since birth, there are marked differences in the occurrence of RV failure and clinical outcome. In patients with pre-capillary pulmonary hypertension (PH), the pulmonary vascular resistance and pulmonary arterial pressure increase more gradually than in massive pulmonary embolism. This gradual increase allows time for RV remodeling and hypertrophy, adaptive mechanisms aimed at decreasing afterload for the RV and maintaining cardiac output. In most PH patients these adaptive mechanisms fall short and, unless treated successfully with pulmonary vasodilator therapy, RV failure, and subsequent death are common within a few years after diagnosis.[2] In patients with chronically increased RV pressure related to a CHD, resulting from either high vascular resistance or RV outflow tract obstruction, this increased pressure has been present since birth. The RV in these patients is often capable of generating high pressures and maintaining cardiac output for many decades.[3,4] However, survival and time to RV failure also differ substantially between patients with pulmonary valvular stenosis (PS), in whom RV failure is rare, and patients with an RV facing systemic vascular resistance.[3,4]

Previous studies have shown that the prognosis of all these patient groups is dependent on the adequacy of RV adaptation.[5–7] Hence, differences in RV adaptation likely explain the clear differences in their clinical course.[8] As yet, RV adaptation in different pressure-loaded conditions are incompletely understood and have not been studied using cardiac magnetic resonance imaging (CMR).

The current study compares RV adaptation in three distinct patient populations: pre-capillary PH, systemic RV, and PS patients–to each other and healthy controls. RV remodeling and RV function were characterized by CMR and echocardiography, taking into account the different components of RV contraction. The study aims to improve the understanding of RV adaptation to chronically increased pressure, through a detailed assessment of RV remodeling, and the different components of RV contraction.

## Methods

This cross-sectional cohort study prospectively enrolled study participant between August 2012 and November 2013, in three tertiary referral hospitals. The Ethical Review boards of all participating centers approved the study and written informed consent was obtained from all participants prior to inclusion. The primary ethical review committee was at University medical Center Utrecht (number METC-11-003). The ethical review boards of both Radboud University medical Center (number 2011/329) and Antonius Hospital Nieuwegein (number LTME/L-11.34/RV-def) also extended their approval. All investigations were performed in one centre (UMCU). Investigations were done consecutively, with less than 30 minutes between CMR and echocardiography. The study protocol conformed to the ethical guidelines of the 1975 Declaration of Helsinki.

### Inclusion and exclusion criteria

Included were adult patients (≥18 years) with a diagnosis of pre-capillary PH (idiopathic or inoperable thrombo-embolic pulmonary hypertension), PS, or RV in systemic position (either congenitally corrected transposition of the great arteries or after atrial switch procedure). Patients had no associated congenital heart defects. Patients with PS were considered for inclusion if stenosis was moderate or severe according to ESC guidelines.[9] The PS-group consisted of patients with native valvular pulmonary stenosis or patients with a residual gradient (moderate or severe) after previous intervention for severe PS. Patients with moderate or severe pulmonary valve regurgitation were excluded. Right heart catheterization had previously confirmed the diagnosis of pre-capillary PH.[10] At the time of study entry, PH patients were still required to fit the criteria for “high echocardiographic probability of PH” as defined by the 2015 ESC guideline PH.[10]

The treating physician determined therapeutic regimen in each patient. All PH patients were on PH-specific therapy and approached if they were clinically stable during their last outpatient clinic visit. Patients with overt clinical RV heart failure, i.e. signs of fluid retention on physical examination, were excluded.

A group of healthy volunteers, free of known disease, aged 18–60 years, were recruited to serve as a reference population. Physical examination, medical history, and electrocardiogram were used to screen volunteers. If these investigations or subsequent CMR and echocardiogram showed abnormalities, they were excluded.

The study protocol conformed to the ethical guidelines of the 1975 Declaration of Helsinki and the human research committees of all recruiting centres approved the study. All participants provided written informed consent prior to enrolment.

### RV systolic pressures

Right ventricular systolic pressure (RVSP) was calculated using the simplified Bernoulli equation, using the maximum velocity of tricuspid regurgitation, or by using the pulmonary valve gradient (in PS patients), both also including the estimated right atrial pressure.[11] In systemic RV patients, the systemic systolic blood pressure at rest was used as RVSP. We estimated end-systolic RV wall stress using an adaptation of Laplace’s law. To take into account different RV geometries and eccentric RV remodelling [12,13], end-systolic volume (RVESV) and RV mass were used instead of diameters, in the following formula: Estimated global RV wall stress = RV systolic pressure x RVESV x 0.5 / RV mass.

### CMR sequence

Steady-state free precession cine images were acquired during end-expiratory breath holds on a commercially available 1.5-T system (Ingenia R4.1.2; Philips Healthcare, Best, the Netherlands). Multi-slice cine short-axis acquisition was planned from the apex up to and including the atrioventricular valves or basal bulge of the RV (TR/TE 3.4/1.69 ms, voxel size 1.3x1.3x8.0mm, flip angle 55^o^; 30 phases per cycle). Quantitative through-plane flow (Q-flow) was measured at the level of the pulmonary valve with a retrospectively ECG gated, velocity-encoded phase-contrast sequence (TR/TE 5.2/3.1 ms, voxel size 2.5x2.5x8mm, flip angle 12^o^, field of view 320, matrix 128x100, 20 phases per cycle).

#### CMR: RV volumetrics & geometry

RV volumetric analysis was performed by manually tracing the endocardial and epicardial contours in the end-diastolic and end-systolic phases of all slices, using Qmass MR Research edition (version 7.4, Medis, Leiden, the Netherlands). If the end-diastolic or end-systolic phase were not clear from visual assessment, multiple phases were contoured. The database included the respectively largest or smallest volume. Trabeculae and papillary muscles were excluded from the blood volume.[14] The following parameters were measured: RV end-diastolic volume (EDV), RV end-systolic volume (ESV), RV ejection fraction (RVEF), RV mass and RV mass-volume ratio. Q-flow measurements (Medis Qflow, version 5.5, Medis, Leiden, the Netherlands) were used to measure the effective RV stroke volume (SV) and cardiac output (RVSV*heart rate). All volumetric data were indexed for Body Surface Area.

**Fig 1:**
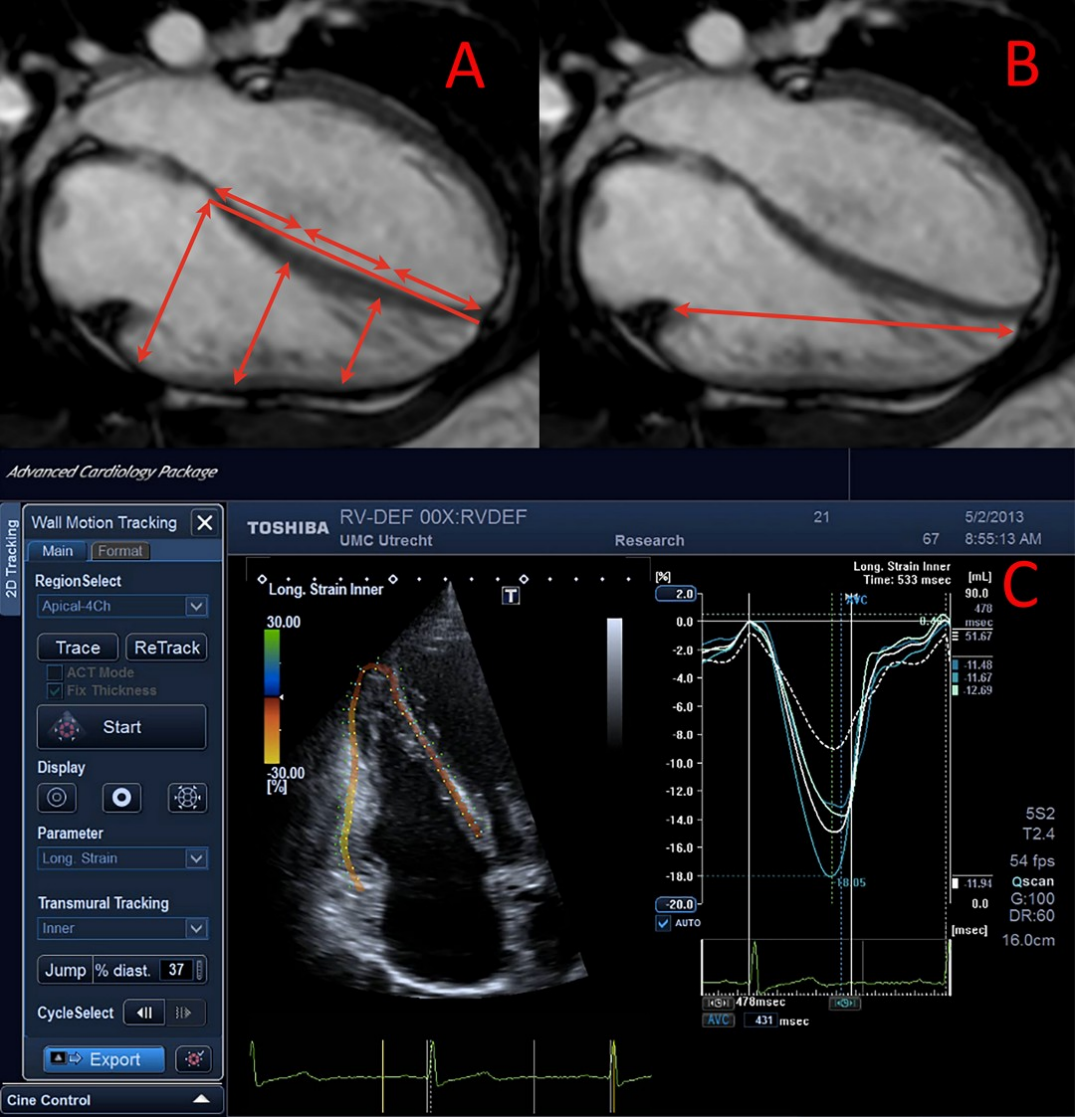
An example of cardiac magnetic resonance and echocardiographic analyses. A&B: Image of CMR analysis for transverse motion (a) perpendicular to the apex-base axis and of longitudinal motion (b). C: Image of longitudinal deformation analysis of the RV.

RV geometry, transverse motion, and longitudinal free wall motion were measured on steady-state free precession cine images in the 4-chamber orientation, as indicated in Fig 1. Systolic dimensions were measured at maximum RV free wall thickening before potential rapid septal displacement. The mid to basal and apex to basal ratios, transverse and longitudinal motion (diastolic-systolic dimension) and shortening ([diastolic-systolic dimension]/diastolic dimension) were calculated.

### Echocardiography: Longitudinal deformation

Echocardiography was performed on a Toshiba Artida system (Tokyo, Japan) with a 5-MHz transducer. Echocardiographic RV measurements were taken in an RV–centric 4-chamber view (example in Fig 1), including off-line longitudinal speckle tracking analysis (Advance Cardiac package version 3.0, Toshiba). Peak *systolic* strain and post-systolic-shortening ([peak strain–peak *systolic* strain]/peak strain) were measured for the RV free wall, the interventricular septum (IVS) and global RV strain. Pulmonic valve opening, acceleration time, pulmonic valve closure, and ejection time were measured from a pulsed wave Doppler tracing taken in the RV outflow tract. Measurements were taken at an inter-beat (RR) interval similar to that of the strain analysis. Two measures of RV dyssynchrony were included: the time difference between peak septal and peak free wall strain, and the standard deviation of the basal-RV free wall, mid-RV free wall, basal septal, and mid septal segment time to peak (RV-SD4). [15]

### Exercise testing

Exercise testing was performed using an electronically braked cycle ergometer using a ramp protocol, at a cadence of 70 revolutions per minute until exhaustion. Peak oxygen uptake per kilograms bodyweight (pVO2/kg), peak oxygen uptake per heart rate (pVO2/HR), peak heart rate (HR), and VE/VCO2 slope were measured and expressed as % of predicted for age, sex and weight. To account for the disparity in BMI the normative values were derived from Koch et al.[16] As this study did not include patients <25 years of age, we used normative values from Jones et al. for this age group.[17]

### Statistical analysis

All continuous variables are presented as mean ± standard deviation or median [range] as appropriate. Categorical variables are shown as frequency (%) and compared with Chi-square. All patient groups were compared to controls using Analysis of Variance (ANOVA) with posthoc Dunnet’s–for normally distributed parameters–or Kruskal-Wallis with Mann-Whitney U test–for non-normally distributed parameters. Patient groups were compared to each other using Analysis of Variance (ANOVA) with posthoc Bonferroni.

The Pearson correlation coefficient (r) was used to correlate conventional echocardiographic measurements, deformation imaging and transverse measures to RVEF. Multivariable linear regression analysis included all imaging parameters that were correlated significantly with RVEF. If parameters were suspected to be collinear, the multivariable analysis only included the parameter with the highest univariable correlation. Multivariable linear regression, including underlying disease as an additional variable, was also used to associate the same measurements to % of predicted VO2max/kg. For multivariable linear regression, we report the adjusted R^2^ for the model and beta-coefficient for the individual parameter.

Intra- and interobserver reproducibility were assessed by re-analyzing 15 datasets >1 month apart. The agreement was assessed using intra-class correlation coefficients, mean difference with limits of agreement and a paired Student T-test. Our group and Kind et al. have previously published reproducibility of the CMR measurements. [14,18] An alpha level of 0.05 was considered statistically significant.

## Results

The study included 91 subjects: 37 controls, 15 PS, 19 systemic RV (15 patients post-atrial switch and four congenitally corrected transposition of the great arteries), and 21 PH patients (9 idiopathic & 12 chronic thrombo-embolic; median 4.8 [0.6–35.0] yrs since diagnosis). Table 1 lists all demographic data. All patients were in atrial or sinus rhythm. PH patients were on the following PH-specific medication: prostacyclin 4/21 (19%), PDE5-inhibitor 14/21 (67%), and endothelin-receptor antagonist in 19/21 (90%).

**Table 1:** Patient characteristics. Data are presented as mean±standard deviation, compared using ANOVA and posthoc Dunnet’s, or median [range], with Kruskal-Wallis and Mann-Whitney U test.

	Controls (n = 37)	PS (n = 15)	Syst RV (n = 19)	PH (n = 21)
**Age (yrs)**	33.2 [21.0–60.2]	25.5 [18.2–43.3]	32.7 [28.4–52.9]	54.3 [19.5–74.0]‡
**Male (%)**	22 (59.5%)	4 (26.7%)*	13 (68.4%)	6 (28.6%)*
**BSA (m**^**2**^**)**	1.89±0.20	1.86±0.14	1.94±0.14	1.89±0.20
**BMI (kg/m**^**2**^**)**	23.3±2.4	24.7±2.4	26.4±5.5	27.7±6.6†
**Medication:** **- Beta-blocker** **- ACE-inhibitor** **- Diuretics (any kind)**	- - -	- - -	7 (36.8%) 5 (26.3%) 2 (10.5%)	1 (4.8%) 3 (14.3%) 15 (71.4%)
**QRS_dur (ms)**	96 [66–114]	96 [78–148]	100 [70–130]	80 [70–140]
**RBBB**	0 (0%)	2 (3%)	5 (26.3%)	1 (4.8%)
**QTc (ms)**	401±22	420±32	419±33	408±42
**RVSP (mmHg)**	18.1±3.3	56.0±15.7	110.8±11.8	66.7±16.7
**None/trace TR** **Mild TR** **Moderate TR** **Severe TR**	37 (100%) - - -	9 (60%) 5 (33%) 1 (7%) -	3 (16%) 12 (63%) 3 (16%) 1 (5%)	5 (24%) 9 (43%) 6 (29%) 1 (5%)
**Performance measurements**				
**NYHA I** **NYHA II** **NYHA III**	37 (100%) - -	15 (100%) - -	17 (89.5%) 2 (10.5%) -	1 (4.8%) 16 (76.2%) 4 (19.0%)
**pVO2/kg**^**a**^	-	30.1±7.8	26.3±6.4	16.5±4.9
**%pVO2/kg**^**a**^	-	92.2±16.9	80.8±16.9	62.4±15.5
**VE/VCO2 slope**	-	26.2±5.8	27.7±3.9	41.5±10.3
**% pred VO2/HR**	-	97.6±17.7	96.7±27.3	72.3±16.9
**% pred HR peak**	-	100.3±6.9	87.9±11.8	89.8±10.5

*P<0.05

†p<0.01 and

‡p<0.001.

^a^ missing in 3 PH patients due to chronic oxygen use

Syst = systemic; BSA = Body surface area; BMI = body mass index; pVO2/kg = peak oxygen uptake per kilogram body weight (ml/kg/min); %pVO2/kg = percentage of predicted; VE/VCO2 = minute ventilation relative to carbon dioxide elimination; %pred VO2/HR = oxygen uptake/peak heart rate

RVSP was significantly higher in systemic RV patients than PH and PS patients (p<0.001) but did not differ significantly between PS and PH patients (p = 0.111). Percentage of predicted pVO2/kg and pVO2/heart rate were significantly lower in PH patients than PS and systemic RV patients (both p<0.01). VE/VCO2 slope was significantly higher in PH patients (both p<0.001). Lastly, % of predicted peak HR was lower in PH patients and systemic RV patients compared to PS patients (respectively p = 0.003 and p = 0.015).

### RV size and mass

**Fig 2:**
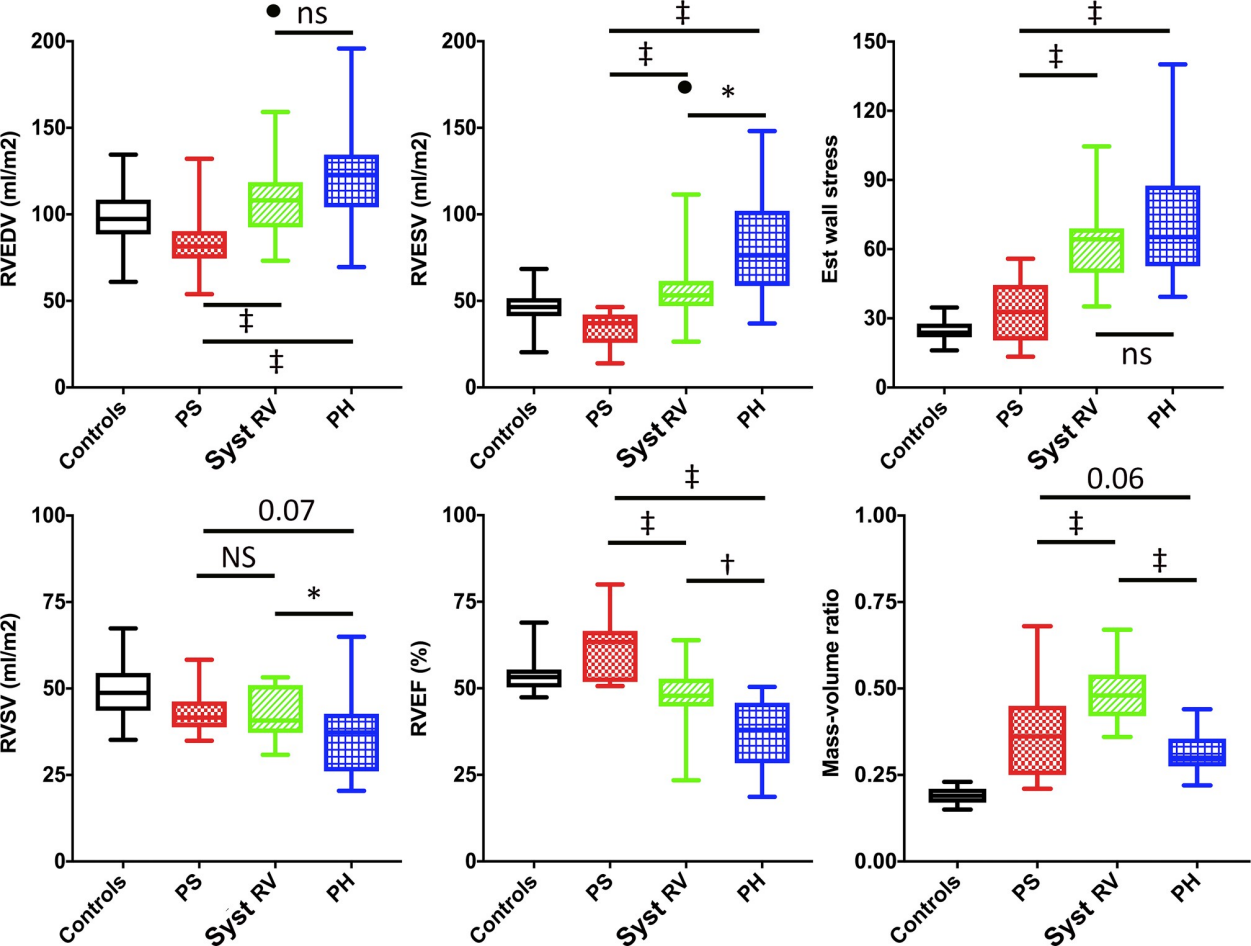
CMR measurements. 2a: RV end-diastolic (EDV), end-systolic volume (ESV) and estimated wall stress; outlier in systemic RV group (•). 2b: RV stroke volume (RVSV), RV ejection fraction (RVEF) and mass-volume ratio. Patient groups were compared using ANOVA with posthoc Bonferroni. *p<0.05; †p<0.01; ‡p<0.001. PH = pulmonary hypertension; PS = pulmonary stenosis; Syst RV = systemic right ventricle.

**Fig 3:**
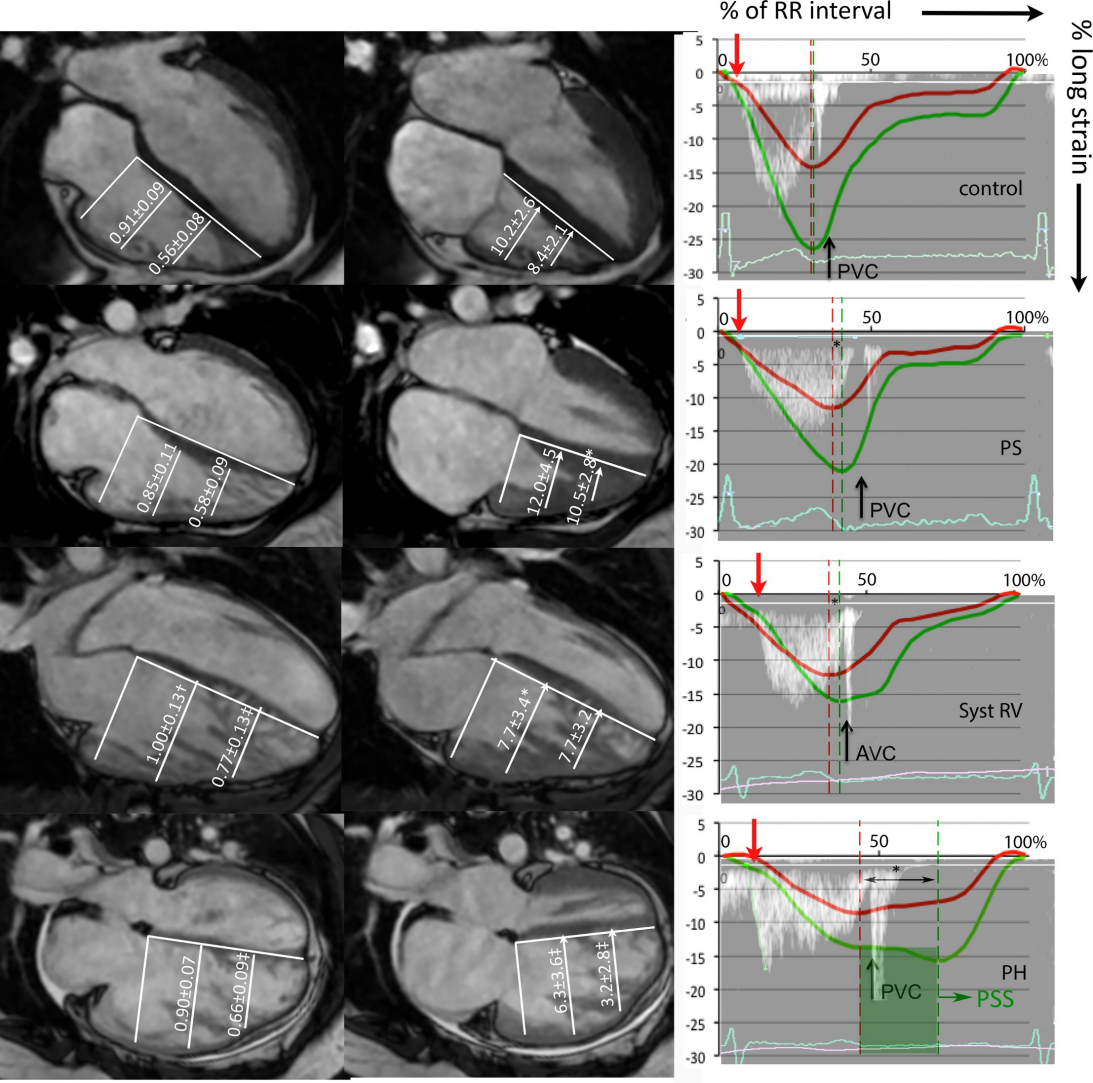
RV geometry, transverse motion, and timing of contraction. Left column: RV mid-basal and apical-basal ratio. Mid column: RV transverse motion at mid and apical level. The right column: timing of RV free wall (green) strain, septal (red) longitudinal strain, and RVOT Doppler tracing from a representative patient presented as percentage (0–100%) of the RR interval. Pulmonary valve opening (red arrow), pulmonary valve closure (PVC) and the difference between septal and free wall peak (*) are also shown. Patients were compared to controls using ANOVA with posthoc Dunnet’s; *p<0.05; †p<0.01; ‡p<0.001. PS = pulmonary stenosis; Syst RV = systemic righ ventricle; PH = pulmonary hypertension.

Table 2 and Fig 2 detail RV volumetrics and mass. RV volumes were decreased in PS patients compared to controls (p<0.01). Conversely, systemic RV (p<0.05) and PH patients (p<0.001) had increased RV volumes. As shown in Fig 3, systemic RV and PH patients also have more spherical RV geometry compared with controls (Fig 3) and PS patients (p<0.002 apical-base ratio).

**Table 2:** CMR measurements. Data are presented as mean±standard deviation, groups were compared using ANOVA and posthoc Dunnet’s. Non-normally distributed data presented as median [range], groups were compared using Kruskal-Wallis and Mann-Whitney U test.

Volumetric data	Controls (n = 37)	PS (n = 15)	Syst RV (n = 19)	PH (n = 21)
**LVEDV (ml/m**^**2**^**)**	97.3±13.7	84.6±10.3*	79.6±12.1‡	74.6±12.1‡
**LVEF (%)**	55.4±3.5	57.3±4.0	57.9±7.9	55.9±7.8
**RVEDV (ml/m**^**2**^**)**	97 [61–135]	81 [54–132]†	110 [73–222]*	123 [70–196]‡
**RVEF (%)**	53.8±4.5	61.1±9.6*	46.3±10.1†	36.9±9.6‡
**RV mass (g/m**^**2**^**)**	18.5±3.7	29.3±11.0‡	54.9±13.5‡	37.9±10.9‡
**RV cardiac output (l/min)**^**a**^	5.7±1.2	5.4±0.8	4.7±0.8*	4.7±1.6†
**Geometric data**				
**Longitudinal motion (mm)**	23.5±3.2	20.0±3.0†	14.6±3.2‡	15.4±3.6‡
**Trans shortening apex(%)**	31.5±7.5	39.6±13.6*	17.4±6.9‡	9.3±7.7‡
**Trans shortening mid (%)**	23.4±5.7	30.0±10.9	13.4±6.3‡	12.4±6.2‡

*P<0.05

†p<0.01 and

‡p<0.001.

^a^Stroke volume and cardiac output missing in 5 participants. Syst = systemic; EDV = end-diastolic volume; ESV = end-systolic volume; EF = ejection fraction; RV = Right ventricular; trans = transverse

### Global RV function and cardiac output

Table 2 and Fig 2 details measurements of global RV function by CMR. PS patients had significantly higher RVEF compared to controls, while systemic RV and PH patients had a significantly lower RVEF. In all patient groups, the indexed RVSV was significantly lower compared with controls (p<0.05 for PS and systemic RV and p<0.001 for PH). However, RV cardiac output was decreased compared to controls only in systemic RV and PH patients (Fig 2).

### Longitudinal and transverse motion

Measurements of longitudinal RV free wall motion, by both CMR (Fig 1) and speckle-tracking echocardiography (STE), were significantly higher in PS patients than in systemic RV or PH patients (all p<0.001; Tables 2 & 3).

**Table 3:** Echocardiographic measurements. All patient groups were compared with controls using ANOVA with posthoc Dunnet’s test or Kruskal-Wallis, as appropriate.

	Controls (n = 37)	PS (n = 15)	Syst RV (n = 19)	PH (n = 21)
**Functional measurements**				
**FAC (%)**^**a**^	40.5±6.6	41.6±8.8	27.9±6.8‡	21.8±8.7‡
**RA area (cm**^**2**^**)**	17.8±3.8	19.2±3.6	19.2±7.5	23.4±6.4‡
**TAPSE (mm)**	23.5±2.9	20.7±5.1*	14.3±3.3‡	19.4±3.7‡
**TDI S’ (cm/sec)**	13.8±1.8	10.7±2.4‡	8.1±3.0‡	11.4±2.5‡
**RVFW long strain (%)**^**b**^	-25.0±2.6	-21.1±5.3	-14.1±4.2‡	-15.5±3.4‡
**IVS long strain (%)**^**b**^	-15.1±1.6	-13.3±2.5	-10.6±2.6‡	-10.4±3.4‡
**RV global long strain (%)**^**b**^	-20.0±1.7	-17.8±3.9	-12.5±3.7‡	-12.8±2.8‡
**Timing of contraction**				
**Heart rate (bpm)**	64±9	67±12	60±11	74±11‡
**Ejection time RVOT (ms)**	320±27	355±33*	289±26*	300±44
**PA acc time (ms)**	154±20	181±28†	130±20‡	77±16‡
**PVO (ms)**	91±15	85±15	117±15‡	100±18
**PVC (ms)**	404±27	441±32‡	406±26	401±41
**TTP RVFW (ms)**^**b**^	399±32	428±25	399±48	453±93†
**RV-SD4 (ms)**^**b**^	27±14	41±21	50±25†	79±29‡
**ΔIVS_RVFW (ms)**^**b**^	-18 [-60; 72]	-49[-103; -25]†	-27[-170; 30]	-73[-273; 35]†

*p<0.05

†p<0.01

‡p<0.001.

^a^Fractional area was available in 78/91 (85.7%) patients.

^b^ Speckle tracking analysis was available in 76/91 (73.6%; median frame rate 57, ranging 42–116 fps).

FAC = fractional area change; RA = right atrial; TDI S’ = Tissue Doppler Imaging systolic velocity of the tricuspid annulus; RV = right ventricle; RVOT = right ventricular outflow tract; PA acc time = acceleration time measured with PW Doppler in RV outflow tract; PVO = pulmonary valve opening; PVC = pulmonic valve closure; RV-SD4 = standard deviation of 4 RV segment time to peak; TTP = time to peak; ΔIVS_RVFW = difference between septal and free wall time to peak.

Fig 3 and in Table 2 detail measurements of transverse motion and shortening. PS patients exhibit increased apical transverse motion compared to controls (p = 0.03). Conversely, apical transverse motion and shortening in PH patients were significantly lower compared with controls (p<0.001), PS (p<0.001) and systemic RV patients (p = 0.02).

### Timing of contraction

Timing measurements are shown in Table 3 and visualized in Fig 3. Time to peak RV free wall contraction is significantly longer in PH compared with controls and systemic RV patients (PS p = 0.09)–continuing after pulmonic valve closure. Post-systolic shortening of the RV free wall was much more frequent in PH patients (n = 5; 45%) compared to controls (n = 1; 7%), PS n = 1 (10%) and systemic RV n = 2 (13%). Post-systolic shortening was <1% in all but 1 CHD patient but ranged from 1.1–14.9% in PH patients. RV-SD4 was higher in PH patients compared to systemic RV (p<0.001) and PS patients (p<0.001). Furthermore, IVS to RV free wall delay was longest in PH patients, but this was not significantly different from PS and systemic RV patients.

### Association of specific imaging markers to RVEF and exercise capacity

Table 4 depicts the correlation between different imaging parameters and RVEF. Fractional area change (r = 0.816; p<0.001), global systolic RV longitudinal deformation by STE (r = 0.812; p<0.001) and apical transverse shortening (r = 0.853; p<0.001) showed the highest correlation to RVEF. PA acceleration time was the timing measurement with the highest correlation to RVEF (r = 0.728, p<0.001). Using multiple linear regression, a combination of global systolic RV longitudinal deformation and apical transverse shortening provided the best model (adjusted R^2^ = 0.828), i.e. together explaining 82.8% of the variation in RVEF.

**Table 4:** Correlation to RVEF. Pearson correlation coefficients of different imaging parameters to RVEF. Multivariable linear regression included parameters with significant correlation coefficient, the model only included the parameter with the highest univariable correlation if colinearity was suspected.

	Pearson r	p-value	Multivariable
**TAPSE (mm)**^**a**^	0.393	0.024	
**TDI S’ (cm/sec)**	0.117	0.368	
**RV-SD4**	-0.482	<0.001	p = 0.794; β = -0.011
**FAC (%)**	0.816	<0.001	p = 0.075; β = 0.286
**RVFW strain (%)**	-0.729	<0.001	
**IVS strain (%)**	-0.723	<0.001	
**RV total strain (%)**	-0.812	<0.001	p<0.001; β = -1.324
**Mid shortening (%)**	0.814	<0.001	
**Apical shortening (%)**	0.853	<0.001	p<0.001; β = 0.588
**PA acc time (ms)**	0.728	<0.001	p = 0.187; β = 0.051
**Ejection time (ms)**	0.521	<0.001	p = 0.514; β = -0.038
**PV closure (ms)**	0.429	<0.001	

^a^Including only patients without previous cardiac surgery. RV = right ventricular; RV-SD4 = standard deviation of time to peak strain for 4 RV segments; FW = free wall; IVS = interventricular septum; PA acc time = acceleration time of pulsed wave Doppler in RV outflow tract; PV = pulmonary valve

Global systolic RV longitudinal deformation by STE was the only parameter associated to % of predicted pVO2max/kg in multiple linear regression (p = 0.025; unstandardized beta coefficient -1.916), independent of underlying disease entity (adjusted R^2^ increasing from 0.349 to 0.392).

### Reproducibility of deformation imaging

Both intra-observer and inter-observer agreement yielded good intra-class correlation coefficients and acceptable limits of agreement (S1 Table).

## Discussion

Differences in clinical outcome between patient groups with chronic RV pressure-overload are well known. However, the differences in RV adaptation that are responsible for this are not well understood. This prospective study is the first to comprehensively compare RV remodeling and function in patients with PS, systemic RV, and PH, using both CMR and echocardiography. The most striking results of the study are twofold: 1) The RVs of patients with PS are hyperdynamic, with low RV volumes and supra-normal RVEF, despite sustaining substantially elevated RV pressures for several decades. 2) In contrast, patients with a systemic RV or with PH show RV dilation, spherical RV geometry, and decreased ejection fraction. Of the latter two patient groups, PH patients have a poorer global function, reflected by a lower RVEF and RV stroke volume. This is probably mediated by a higher degree of intra-ventricular dyssynchrony, resulting in post-systolic shortening, and by decreased transverse RV free wall function. In pressure-loaded RVs apical transverse function and global RV systolic strain by STE were able to predict 83.8% of the variance in RVEF. The correlation of TAPSE and TDI S’, the most frequently used conventional echocardiographic parameters, to RVEF was poor.

### Differences in RV remodeling and global function

Higher systolic ventricular pressure needs to be met by proportional RV hypertrophy to reduce afterload and/or increase contractile strength. If this hypertrophy is sufficient, acceptable stroke volumes can be maintained. If this hypertrophy is insufficient, RV dilation will ensue to maintain stroke volume through the Frank-Starling mechanism: a phenomenon that is known as pre-load recruitable stroke work. If both mechanisms are not sufficient to compensate for the elevated afterload, myocytes remain stretched throughout systole, which increases the duration of contraction and ultimately decreases systolic function.[19]

This theoretically translates into three types of adaptation: 1) increased afterload compensated by adequate RV hypertrophy. 2) Increased afterload compensated by hypertrophy and RV dilation (i.e., using pre-load recruitable stroke work). 3) Increased afterload compensated insufficiently despite hypertrophy and RV dilation, resulting in an increased duration of contraction and a drop in stroke volume (ventriculo-arterial uncoupling). Patients with valvular stenosis have low RV volumes, high mass-volume ratios and (supra)normal function, corresponding to the first pattern of adaptation. The RV of systemic RV patients and PH patients, facing high vascular resistance, dilates to maintain its stroke volume (Fig 2). Systemic RV patients can maintain adequate RVEF and RVSV, without prolongation of RV free wall contraction. This indicates that their RVs are still able to compensate for the increased afterload (pattern 2), which is likely enabled by the higher mass-volume ratio in the systemic RV group. Contrarily, PH patients show poor global function, a drop in stroke volume, and prolonged duration of contraction—but not of ejection (Table 3). These findings are likely signs of stretch on the myocytes throughout systole, [19] indicating insufficiently compensated afterload (pattern 3). [13,20]

### Components of RV contraction

Measures of RV free wall longitudinal shortening, which is believed to be most important for global RV function in the normal RV, were decreased in all patient groups compared to controls (Table 3). In patients with pressure-loaded RVs other components of RV contraction–i.e., radial strain, circumferential strain, and the transverse component of the bellows effect[21]–may become more important for overall RV performance. To investigate these components, we measured the transverse motion of the RV free wall as a surrogate marker. Indeed, PS patients showed increased transverse motion compared to controls, patients with systemic RV, and patients with PH, which contributes to the supranormal RVEF in this group. Furthermore, RVEF in PH patients was decreased compared to systemic RV patients, despite comparable RV free wall longitudinal motion and strain. The reduced transverse RV free wall motion in PH patients can in part explain the lower RVEF. The importance of transverse motion for RV assessment in PH patients is underlined by previous studies, which have related the transverse component of contraction to survival in mixed populations with PH. [6,22] Furthermore, transverse shortening was closely related to RVEF in our cohort of patients with RV pressure-overload (r = 0.853), underlining its importance for global function in the pressure-loaded RV.

### Timing of contraction

Increased intra-ventricular dyssynchrony mediated by prolonged RV free wall contraction, present in PH but not CHD patients, is a second important mechanism of RV dysfunction in PH patients.[20] The RV free wall longitudinal contraction in PH patients continues beyond pulmonary valve closure and septal contraction (Fig 3), leading to post-systolic shortening and rapid leftward septal motion. This results in highly inefficient RV contraction (as visualized in Fig 3) and compromises LV diastolic filling, which is reflected by the decreased LVEDV in PH patients (Table 2).[8,13,20] This intra-ventricular dyssynchrony cannot be explained electromechanically, as QRS duration was similar between the groups and RBBB was infrequent. As discussed above, the prolonged contraction is probably a sign of insufficiently compensated afterload in PH patients, causing a mechanical RV free wall delay. Furthermore, PH patients also showed a larger dispersion in regional time to peak, as measured using RV-SD4. As previously demonstrated in PH patients, this may relate to regional differences in wall stress and eccentric RV remodeling.[23]

### Why is adaptation different?

The ultimate goal of RV adaptation is for the RV to stay coupled to its afterload.[24] The differences in RV adaptation between CHD and PH may be the result of differences in ventricular load, adequacy of RV remodeling, and/or ability to increase myocardial contractile force (by intrinsic myocardial properties).

The ventricular load is most often estimated using calculations of end-systolic wall stress (static load), but oscillatory load (i.e., vascular stiffness, compliance and reflected waves) also determines the total RV load.[25] Although static load was probably is highest in systemic RVs, oscillatory load is increased in PH.[25,26] The relative contributions of static and oscillatory load to the total hydraulic load are not completely understood, but differences in type of afterload likely contribute to the observed differences in RV adaptation.

Furthermore, the myocardial response, regarding RV hypertrophy, differs substantially between groups. Both CHD patient groups show superior mass-volume ratios compared to the PH patients, indicating a higher degree of hypertrophy, which has been reported in previous studies as well.[8,27] This may be explained by a different time of onset, as the RV exposed to abnormal hemodynamics from fetal life onwards (in CHD but not PH) may maintain its “fetal” phenotype–with a thick RV myocardium and possibly favorable myocardial properties.[28,29]

Lastly, myocardial damage might progress faster in PH, as studies have demonstrated relative RV ischemia, causing fibrosis–which aggravates RV dilation and reduces contractility. [30]

### Practical considerations

CMR-derived RVEF is considered the reference standard for assessment of RV systolic function. However, it is expensive, not very patient-friendly, and therefore, not used as the sole imaging modality for follow-up in these patients. In daily practice, most institutions will use echocardiography for serial follow-up. The echocardiographic measurements most frequently used for quantification of RV systolic function are TAPSE, TDI S’ and fractional area change. In accordance with recent studies, correlation of TAPSE to RVEF was poor in our study.[31,32] Fractional area change was the only “conventional” echocardiographic measurement that showed an excellent correlation (r = 0.816) to RVEF–as previously demonstrated in PH patients.[33,34] Both global RV systolic longitudinal deformation by STE, which is the composite of RV free wall and septal strain, and transverse shortening (which can also be measured using echocardiography) also showed excellent correlation to RVEF (Table 4). Combining both measurements improved the predictive value of the regression model to R^2^ = 0.83. Their value in daily practice will require further study, as they are less simple and straightforward than TAPSE and fractional area change. Our results complement previous studies in PH, showing global RV strain[34,35] and transverse motion[33] are of added value for assessment of RV systolic function in a mixed cohort with RV pressure-load.

PA acceleration time also correlated well to RVEF, with a shorter acceleration time corresponding to a poorer RVEF. The fact that not RVSP but PA acceleration time correlated to RVEF may indicate acceleration time–like the pulsed wave Doppler shape–better reflects the interaction between ventricular and pulmonary vasculature (i.e., ventricular-arterial coupling).[36]

Global longitudinal deformation by STE was the only measurement related to % of predicted VO2max/kg independent of underlying disease. Different determinants of aerobic exercise capacity in CHD vs. PH patients likely explain this. In patients with CHD, aerobic capacity is mainly related to cardiac output, either a decreased O2 pulse (representing reduced stroke volume) or–as seen in the systemic RV group–reduced peak HR. Contrarily, in PH patients both cardiac function and decreased ventilatory efficiency contribute to the decreased aerobic capacity [15], as illustrated by the high VE/VCO2 in Table 1.

### Limitations

No invasive pressure measurements were available–as they are not routinely performed in many CHD patients. Furthermore, RVSP was inevitably higher in systemic RV patients. However, our goal was to have comparable RVSP in PH and PS patients. As all imaging measurements of RV function are load dependent, it is important to note that differences in ejection/deformation do not always reflect contractility, but may be a result of different afterload.

Although we attempted to reduce heterogeneity to a minimum, sex and age distribution were different between the groups, concordant with the differences in epidemiology. However, it is unlikely that the differences in age importantly influenced our results, as the normal effect of age on RV function and volume is opposite to the change observed in PH patients.[37] Lastly, some of the PS patients had previously undergone an intervention for severe PS. As patients presenting with severe PS during adulthood are rare in Western countries, most will have had intervention following current guidelines.[9] The objective was to include PS as a patient group with elevated RV pressures and an RV in the normal subpulmonary position. To compare them to PH patients, we excluded PS patients with moderate or severe regurgitation. Although the results might have been stronger if the study only included patients without previous interventions, the differences in RV function and RV remodeling are still clearly shown.

There is no agreed-upon way to calculate wall stress for the RV. We estimated end-systolic wall stress with a modified Laplace’s law; using wall mass and end-systolic volumes to obtain a more global estimate of wall stress as RV geometry varies greatly between patient entities.

## Conclusion

Patient groups with high RV pressures greatly differ in presence and degree of RV dilation, RV mass to volume ratio, magnitude of transverse motion, and timing of RV contraction–which corresponds to the degree of RV dyssynchrony. This ultimately leads to differences in stroke volume and global function. Concurrent to their poor clinical outcome, PH patients showed unfavourable RV remodeling and function. This indicates that their RV, unlike that of PS and systemic RV patients, is unable to compensate for its increase in afterload completely. In the studied cohort with RV pressure overload, global RV systolic longitudinal deformation by STE, transverse motion, and fractional area change correlated highly to RVEF, while TAPSE and TDI S’ did not.

## Supporting information

S1 Table Reproducibility of longitudinal deformation. Reproducibility measurements. ‡mean difference and limits of agreement.; ‡mean difference and limits of agreement; II significance of paired T-test. ICC = intra-class correlation coefficient; RV = right ventricular.
(DOCX)

S1 Data Anonymized dataset. (SAV)
