# Supplementary material for: RV adaptation to increased afterload in congenital heart disease and pulmonary hypertension
Source: PLoS One. 2018 Oct 24;13(10):e0205196. doi: 10.1371/journal.pone.0205196 (PMC6200212; doi:10.1371/journal.pone.0205196)
Supplement: S1 Table — Reproducibility measurements. ‡mean difference and limits of agreement.; ‡mean difference and limits of agreement; II significance of paired T-test. ICC = intra-class correlation coefficient; RV = right ventricular. (DOCX) [file pone.0205196.s001.docx]

**S1 Table**

|  | **ICC** | **p-value** | | **Absolute mean Δ‡**  **[limits of agreement]** | **p-value^II^** |
| --- | --- | --- | --- | --- | --- |
| **Intra-observer** |  | |  |  |  |
| **RV free wall**  **Septal**  **RV total** | 0.98  0.85  0.95 | | <0.001  <0.001  <0.001 | -0.18% [-1.96; 1.60]  -0.18% [-2.64; 2.29]  -0.06% [-2.08; 1.94] | 0.471  0.600  0.850 |
| **Inter-observer** |  | |  |  |  |
| **RV free wall**  **Septal**  **RV total** | 0.87  0.77  0.91 | | <0.001  <0.001  <0.001 | 2.04% [-1.29; 5.37]  1.02% [-1.43; 3.47]  1.14 % [-0.96; 3.24] | 0.004  0.016  0.008 |
